# Supplementary material for: Nr1d1 Mediated Cell Senescence in Mouse Heart-Derived Sca-1+CD31− Cells
Source: Int J Mol Sci. 2022 Oct 18;23(20):12455. doi: 10.3390/ijms232012455 (PMC9603916; doi:10.3390/ijms232012455)
Supplement: Supplementary file 1 [file ijms-23-12455-s001.zip › Figure S1,2,3.pdf]

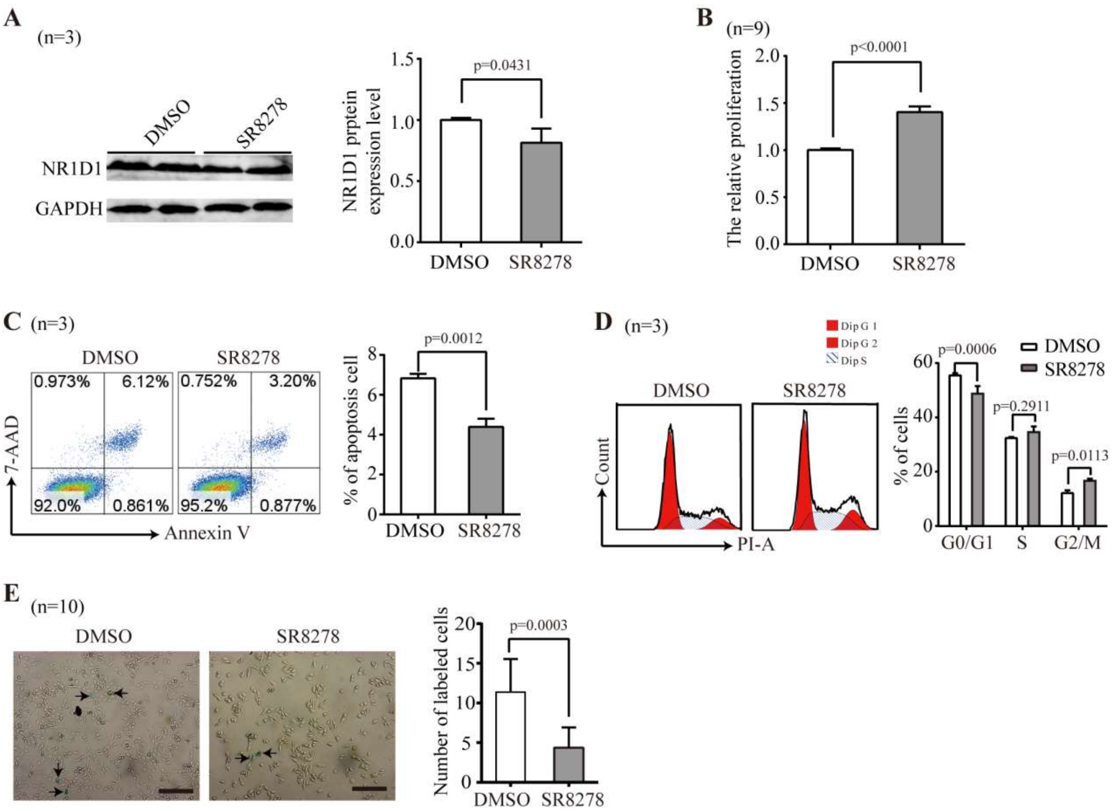

Supplementary Figure S1. Effect of inhibition of *Nr1d1* expression on cellular senescence.

(A-E): MCM was treated with SR8278, and the control was treated with DMSO. (A) Immunoblotting of NR1D1 protein levels in MCM cells, data were from three independent experiments(n=3). Two-tail unpaired T test was applied for statistical analysis, $p<0.05$ was considered significant. (B): MEM proliferation was detected by MTT assay, data were from three independent experiments, each experiment had 3 replicates(n=9). Two-tail unpaired T test was applied for statistical analysis, $p<0.05$ was considered significant. (C): Apoptosis of MEM detected by annexin V-PE/7-AAD. The left panel is a representative flow cytometric scatter plot, and the right panel is a graph of apoptosis statistics, data were from three independent experiments(n=3). Two-tail unpaired T test was applied for statistical analysis, $p<0.05$ was considered significant. (D): Cell cycle analysis in MCM performed by FCM, data were collected from three independent experiments(n=3), two-way ANOVA was applied for statistical analysis and $p<0.05$ was considered significant. (E): Analysis of Senescence-associated beta-galactosidase staining in MCM. The image on the left is a representative field of view, scale bars: 50 μm. The graph on the right shows the number of cells that were stained blue in each random field of view. Each experiment was repeated 3 times, and 10 random fields of view were counted(n=10). Two-tail unpaired T test was applied for statistical analysis, $p<0.05$ was considered significant. All data in bar graph presented as mean ± SD.

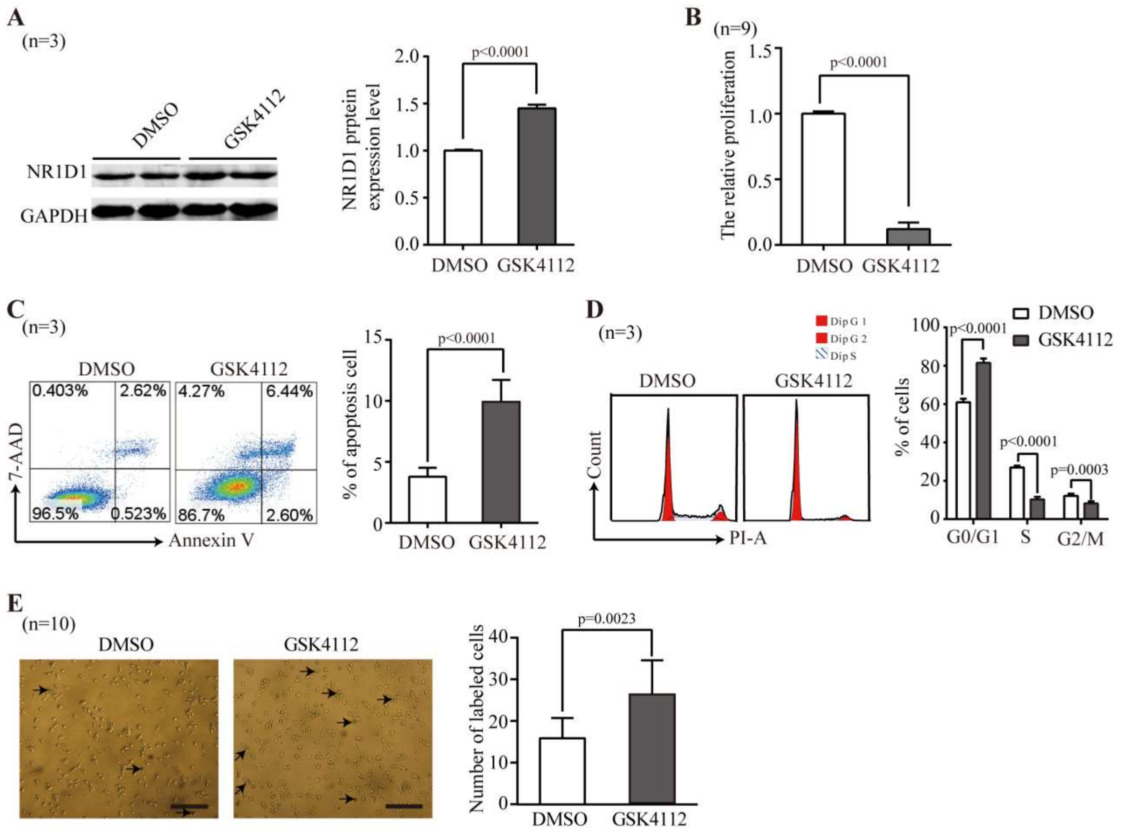

Supplementary Figure S2. Effect of promoting *Nr1d1* expression on cellular senescence.

(A-E): MCM was treated with GSK4112, and the control was treated with DMSO. (A) Immunoblotting of NR1D1 protein levels in MCM cells, data were from three independent experiments(n=3), two-tail unpaired T test was applied for statistical analysis, $p<0.05$ was considered significant. (B): MEM proliferation was detected by MTT assay, data were from three independent experiments, each experiment had 3 replicates(n=9), two-tail unpaired T test was applied for statistical analysis, $p<0.05$ was considered significant. (C): Apoptosis of MEM detected by annexin V-PE/7-AAD. The left panel is a representative flow cytometric scatter plot, and the right panel is a graph of apoptosis statistics, data were from three independent experiments(n=3), two-tail unpaired T test was applied for statistical analysis, $p<0.05$ was considered significant. (D): Cell cycle analysis in MCM performed by FCM, data were collected from three independent experiments(n=3), two-way ANOVA was applied for statistical analysis and $p<0.05$ was considered significant. (E): Analysis of Senescence-associated beta-galactosidase staining in MCM. The image on the left is a representative field of view, scale bars: 50 μm. The graph on the right shows the number of cells that were stained blue in each random field of view. Each experiment was repeated 3 times, and 10 random fields of view were counted(n=10), two-tail unpaired T test was applied for statistical analysis, $p<0.05$ was considered significant.. All data in bar graph presented as mean ± SD.

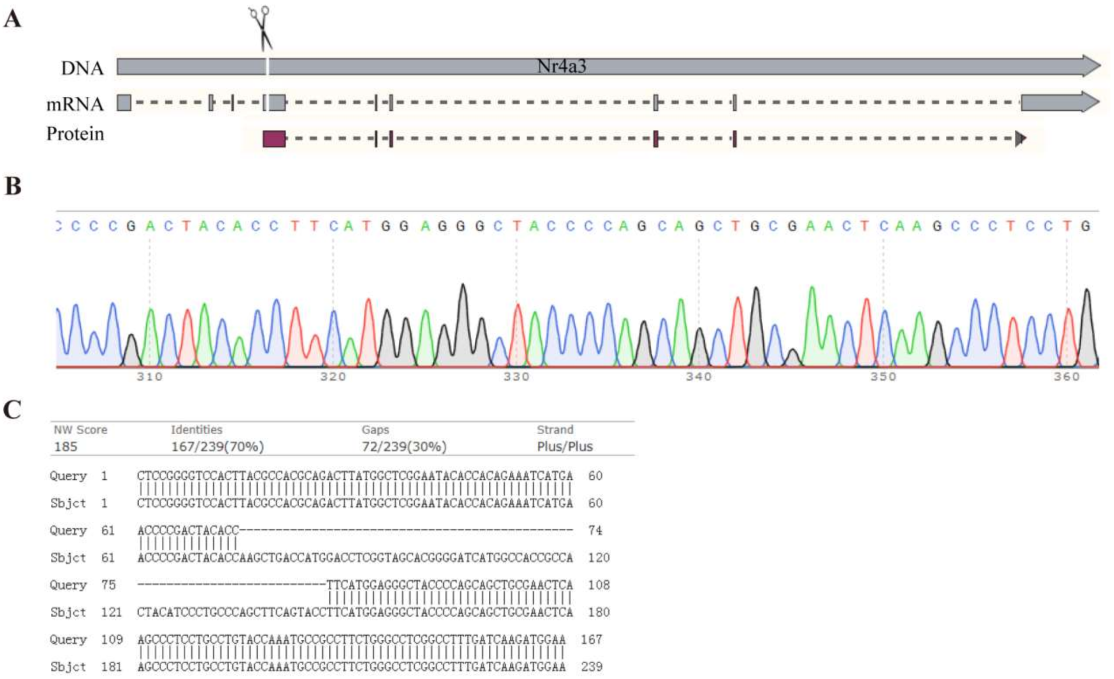

Supplementary Figure S3. Knockout of *Nr4a3*.

(A-C): Knockdown of *Nr4a3* gene in MCM cells by CRISPR/Cas9. (A): Strategies for the *Nr4a3* mutation. (B): Sequencing results of genes near the sgRNA locus after mutation.(C): The deletion of the target gene bases was detected by NCBI-BLAST section.
