# Supplementary material for: Nr1d1 Mediated Cell Senescence in Mouse Heart-Derived Sca-1+CD31− Cells
Source: Int J Mol Sci. 2022 Oct 18;23(20):12455. doi: 10.3390/ijms232012455 (PMC9603916; doi:10.3390/ijms232012455)
Supplement: Supplementary file 1 [file ijms-23-12455-s001.zip › Table S1.pdf]

Table S1: Sequences of primers

Primer	Forward primer 5′ → 3′	Reverse primer 5′→3′
Nr1d1	TGCTTAAGGCTGGCACCTTTG	GTAGGTTGTGCGGCTCAGGAA
Dpt	GAACTATGCCTGCATGCCTACAC	TCTTGCTATAGCGGCAGCAGTAA
Serpina3g	AGGCTGACCTGTCTGCAATCAC	TGCACAACATCCGACACCTG
Nr4a3(for Q-PCR)	CCCAAGTGTACCCAGAGTGGAA	GGAGACTCGGCCATTTGAAAC
Il11	TCGACTGTGACTCGGGACTG	TGTGGGGCGGTTGTTTG
Polr2k	AGATAAAGTCCAGGGATCCCATCAG	CCCACATTTCATCGAGCATCA
Gapdh	GCTGCCCAGAACATCATCCCT	TGAAGTCGCAGGAGACAACC
Nr4a3(for RT-PCR)	GGAGGGAAGATGGATGTAGCC	CGGAGGGAGGAGGAATGGAC
Oligo- *Nr4a3*	caccGACTACACCAAGCTGACCA	aaacTGGTCAGCTTGGTGTAGTC
p-Tianj2	CGAGGGCCTATTTCCCATGAT	GCCAAAGTGGATCTCTGCTG
